# Granulosa cells express three inositol 1,4,5-trisphosphate receptor isoforms: cytoplasmic and nuclear Ca^2+ ^mobilization

**DOI:** 10.1186/1477-7827-6-60

**Published:** 2008-12-09

**Authors:** Mauricio Díaz-Muñoz, Patricia de la Rosa Santander, Anna Berenice Juárez-Espinosa, Rogelio O Arellano, Verónica Morales-Tlalpan

**Affiliations:** 1 Departamento de Neurobiología Celular y Molecular, Instituto de Neurobiología, Campus UNAM-Juriquilla, Querétaro 76230, QRO., México

## Abstract

**Background:**

Granulosa cells play an important endocrine role in folliculogenesis. They mobilize Ca2+ from intracellular stores by a coordinated action between 1,4,5 inositol trisphosphate and ryanodine receptors (IP3R and RyR). The aim of this study was to explore the isoforms of IP_3_Rs expressed in mouse C57BL/6 NHsd granulosa cells, characterizing their intranuclear localization and the relation with other Ca2+-handling proteins.

**Methods:**

Ovarian tissue and granulosa cells were analyzed by multiphotonic and confocal microscopy to determine the intracellular presence of IP3R types 1, 2 and 3, RyR, thapsigargin-sensitive Ca2+-ATPase, and endomembranes. Cellular fractionation and Western blot assays were also used to further confirm the nuclear occurrence of the three IP3R isoforms. Free nuclear and cytosolic Ca2+ concentrations were measured using Fluo-4 AM by confocal microscopy.

**Results:**

By using antibodies and specific fluorophores, was shown that granulosa cells endomembranes contain three isoforms of IP3R, the RyR, and the thapsigargin-sensitive Ca2+-ATPase (SERCA). Interestingly, all these proteins were also detected in the nuclear envelope and in well-defined intranuclear structures. Microsomal membranes depicted characteristic bands of the 3 types of IP3R, but also variants of lower molecular weight. Analysis of nuclear membranes and nucleoplasmic fraction confirmed the nuclear localization of the IP3R types 1, 2 and 3. We demonstrated ATP-induced Ca2+ transients in the nuclear and cytoplasmic compartments. Remarkably, the inhibitory effect on ATP-induced Ca2+ mobilization of brefeldin A was more accentuated in the cytoplasm than in the nucleus.

**Conclusion:**

These findings provide evidence that granulosa cells, including nuclei, express the Ca2+-handling proteins that allow Ca2+ mobilization. All three IP3R were also detected in ovarian slices, including the nuclei of granulosa cells, suggesting that these cells use the three IP3R in situ to achieve their physiological responses.

## Background

Granulosa cells are derived from a keratin-positive epithelium, and function supporting the process oocyte maturation. Granulosa cells are follicular somatic cells and the main source of steroids in the ovary [1,2]. They exert their actions by a combination of paracrine signaling and gap junction-mediated communication [3]. The physiological events characteristic of granulosa cells such as metabolic control, secretion, proliferation, differentiation, and apoptosis, are regulated by numerous factors, but one of the most prominent is the modulation of intracellular Ca^2+ ^concentration ([Ca^2+^]i) [3-7].

Ca^2+ ^is an ionic and biochemical messenger that regulates a great number of cellular functions by acting as a coordinator and effector of metabolic responses among intracellular compartments, such as cytoplasm, endoplasmic reticulum, nucleus, and mitochondria [8]. Ca^2+ ^fulfills its physiological role when: 1) it enters the cell through plasma membrane ion- and receptor-channels, 2) it is released from intracellular stores by ion channels such IP_3_R and RyR, 3) it is extruded from the cell by Ca^2+^/Na^+ ^exchangers and Ca^2+^-ATPases (PMCA) or confined within organelles by others Ca^2+^-ATPases (SERCA), and 4) it is mobilized from or transported into the mitochondria by proton motive force (For review see [9]). Recently, nuclear Ca^2+ ^handling has been the focus of reports which postulate new and original roles in Ca^2+ ^signaling for this organelle, including the presence of invaginations inside the nucleoplasm with the ability to release Ca^2+^[10,11]. Albeit not much information is available regarding the physiological role played by nuclear Ca^2+^, it has been reported that excitation-transcription coupling in myocites is regulated in a nuclear Ca^2+^-dependent manner [12].

Some reports have suggested that this organelle could be acting as an independent and active Ca^2+ ^pool [13]. Accordingly, mechanisms for Ca^2+ ^uptake and release from the nucleus have been recognized in a variety of cells such as neurons, hepatocytes, pancreatic exocrine cells, and starfish oocytes [14]. Ca^2+^-handling proteins, namely IP_3_R, RyR, and thapsigargin-sensitive Ca^2+^-ATPase (SERCA), have been detected in the nuclear envelope [15,16]. Further support for the notion that this organelle can handle Ca^2+ ^by itself are the reports documenting the existence of a nucleoplasmic reticulum in which active IP_3_R, RyR, and SERCA were localized in discrete subnuclear regions [17,18].

Previous reports have established the expression of IP_3_R isoforms in ovarian cells, including granulosa cells [19,20]. Having reported for the first time the expression and subcellular localization of RyR in granulosa cells, and the coordinated activity between RyR and IP_3_R that make possible the ATP-induced Ca^2+ ^mobilization [21], in the present study we further characterize the properties and the type of the Ca^2+^-handling proteins present in these cells. We present experimental evidence that the three isoforms of IP_3_Rs are expressed in the ovarian tissue of C57BL/6 *NHsd *mice. In addition, we demonstrate the presence of all these isoforms in the nuclei of granulosa cells. We also find specific signals in the granulosa cell nuclei using fluorescent probes that recognize RyR, SERCA, and endomembranes. Suggestive evidence of a possible independent Ca^2+ ^handling between compartments was obtained by showing a selective inhibitory action of brefeldin A on cytosolic, but not in the nuclear ATP-induced Ca^2+ ^transients.

## Methods

### Reagents

Insulin, apo-transferrin, penicillin, streptomycin, fetal bovine serum (FBS), Leibowitz medium (L-15), and α-minimal essential medium (α-MEM) were obtained from Gibco BRL (Gaithersburg, MD. USA). Ryanodine, xestospongin C, thapsigargin, and follicle-stimulant hormone (FSH) were from Calbiochem (La Jolla, CA. USA). Fluo-4 AM, BODIPY TR-X Ryanodine, BODIPY-FL thapsigargin, TO-PRO-1 Iodide, brefeldin A BODIPY 558/568 conjugate isomer 1 were obtained from Molecular Probes (Eugene, OR. USA). Sodium pyruvate, 1,4-diazabicyclo [2,2,2] octane (DABCO), paraformaldehyde (PFA), glutaraldehyde, ATP, brefeldin A, bovine serum bovine (BSA), dimethyl sulfoxide (DMSO), the proteinase inhibitors: phenylmethylsulfonyl fluoride (PMSF), aprotinin, leupeptin, pepstatin, and other salts were obtained from Sigma (St. Louis, MO, USA). The Complete mini, Protease inhibitor cocktail tablets was purchased from Roche, (Germany). The NE-PER Nuclear and Cytoplasmic Extraction Reagents Kit was from Pierce (Rockford, IL. USA). The Alkaline Phosphatase (AP) conjugate substrate kit was purchased from Bio-Rad (Hercules, CA. USA). Jung tissue-freezing medium was obtained from Leica (Germany).

Goat polyclonal IgG (immunoglobin G) for isoforms 1, 2, and 3 of the IP_3_R, rabbit anti-goat IgG-Texas Red (TR), rabbit anti-goat IgG AP, and rabbit polyclonal IgG α-actin (H-196) were obtained from Santa Cruz (Santa Cruz, CA. USA). Rabbit anti-goat IgG (H-L) FITC-Conjugate was purchased from ZYMED (San Francisco, CA. USA).

### Cell culture

Cells were obtained based on a published protocol [3,21]. Briefly, C57BL/6 *NHsd *female mice (all animal work was conducted using procedures reviewed and approved by our Institutional animal care, Mexican University), 40–60 days old in different stages of the estrous cycle, were killed by cervical dislocation, and the ovaries were dissected and transferred to a Petri dish with L-15 medium (supplemented with 50 μl/ml FBS, 100 U/ml penicillin, and 100 μg/ml streptomycin). Ovaries were cleaned of neighboring tissue and opened to allow visualization of follicles. Antral follicles (usually 8–10 per ovary) were manually separated from the ovaries and transferred to α-MEM (supplemented with 100 ng/ml FSH, 1 mM sodium pyruvate, 10 μg/ml apo-transferrin, 10 μg/ml insulin, 100 U/ml penicillin, and 100 μg/ml streptomycin). Each follicle was opened using fine forceps, and the granulosa cells were carefully removed and transferred to freshly prepared α-MEM, disaggregated mechanically, and placed on glass coverslips coated with poly-D-lysine. The primary culture was maintained in an incubator at 37°C and 5% CO_2 _for 2–3 days.

### Ovary histology

In independent experiments, female C57BL/6 *NHsd *mice were anesthetized and perfused by intracardiac puncture with phosphate buffer (PBS in mM: 2 KH_2_PO_4_, 3 KCl, 10 Na_2_HPO_4_, 140 NaCl, pH adjusted to 7.4 with NaOH, containing 40 μl/ml PFA) for 8–10 min. Subsequently the mice were decapitated; and the ovaries were removed and cleaned to eliminate the adjacent tissue, then incubated in PBS-40 mg/ml PFA for 4 h at room temperature. Isolated ovaries were transferred to PBS containing 300 mg/ml sucrose and incubated for 12 h at 4°C. Finally, the ovaries were put into Jung Tissue Freezing Medium and preserved at -20°C for 12 h. Cryostat sections of 8–10 μm were used. Samples were stored at -80°C until use.

### Immunochemistry

For immunochemical examination, coverslips of granulosa cells were fixed in PBS containing 40 mg/ml glutaraldehyde for 15 min at 40°C and washed three times with PBS. For cryostat sections, the slices were placed for 1 h at room temperature, transferred to 40 μl/ml PFA for 10 min, and washed three times, each for 5 min, with PBS.

Immunodetection of IP_3_R types 1, 2, and 3 was achieved using the method reported in reference [22], with the following modifications: After fixation, the cells were exposed to 50 mg/ml fat-free milk in PBS for 1 h at room temperature (to block protein-binding sites) and washed three times with PBS, each for 5 min. Anti-IP_3_R types 1, 2, and 3 antibodies diluted 1:200 with 50 mg/ml fat-free milk in PBS including 1 μl/ml Triton X-100 (PBST) (to block residual protein-binding sites) were added. The cells were incubated overnight at 4°C and then washed three times with PBS. In order to detect the primary antibody, cells were incubated with rabbit anti-goat IgG-TR or anti-goat IgG (H-L) FITC-Conjugate, diluted 1:1000 in PBST for 1 h at room temperature, and washed six times in PBST. The coverslips were protected with DAPCO, an aqueous mounting medium. Visualization was performed in a confocal microscope with appropriate filters. Images were obtained using a LSM510 laser scanning microscope with a plan apochromatic 63 × oil-immersion objective (numerical aperture = 1.4), and captured at a resolution of 1024 × 1024 pixels.

### Ovarian fractionation for microsomal membranes and Western blotting

Subcellular fractionation of mouse ovary cells was done using the protocol reported by [23]. Briefly, mice were killed by cervical dislocation, and the ovaries were dissected and gently homogenized with a silicon-coated glass homogenizer in SET buffer (containing in mM): 300 sucrose, 1 EDTA, 1, 2-mercaptoethanol, 50 Tris-HCl pH 8.0. The buffer was supplemented with the following peptidase inhibitors: 0.2 mM PMSF and 10 μg/ml each of aprotinin, leupeptin, and pepstatin or Protease inhibitor cocktail tablets. The homogenized tissue was centrifuged at 2,000 g for 10 min to remove residual tissue and heavy particles. The supernatant was recovered and then centrifuged at 105,000 g for 45 min. Microsomal precipitates were diluted in SET buffer. Membranes were kept at -80°C until use. Protein was determined according to the method of Lowry [24].

Membrane fractions were boiled for 10 min and loaded onto 60 mg/ml SDS-polyacrylamide gels. Ovarian proteins were transferred to nitrocellulose membranes using a Mini Trans Blot Semi dry transfer cell (BioRad, Hercules CA). The nitrocellulose membranes were blocked 2 h in PBS-0.5 μl/ml Tween-2 supplemented with 50 mg/ml fat-free milk. After 3 washes with 150 mM NaCl-0.5 μl/ml Tween-20 (NaCl-T), each for 10 min, the membranes were incubated overnight at 4°C with the primary antibody (Goat polyclonal IgG anti-IP_3_R types 1, 2, and 3, dilution 1:250) in PBST supplemented with 1 mg/ml BSA, washed again three times with NaCl-T for 10 min and incubated for 1 h with the secondary antibody (rabbit anti goat IgG-AP, dilution 1:500) in PBST supplemented with 1 mg/ml BSA. After 3 washes with 0.1 M Tris pH 9.5, color associated with the complexes of IP_3_Rs-antibodies was developed using the AP conjugate substrate kit (Bio-Rad, Hercules CA). All fractions were incubated with rabbit polyclonal IgG α-actin (H-196) (1:1000), a rabbit polyclonal antibody raised against amino acids 180–375 of α-actin of human origin. From this point the protocol mentioned in the previous section was followed.

### Nuclear fractionation

Nuclear extracts were obtained according to NE-PER Nuclear and Cytoplasmic Extraction Reagents Kit (complemented with Protease inhibitor cocktail tablets) [13]. Briefly, the isolation of cytoplasmic and nuclear fractions using the NE-PER kit maintains the integrity of the two cellular compartments before extraction. This prevents cross-contamination of proteins between the two fractions. Additionally, we performed a centrifugation (16,000 × g) to obtain nuclear membranes and nucleoplasmic fraction. The protein was calculated according to the Bradford method [25], and analyzed by Western blotting (see previous section).

### Fluorescent probes

Following fixation, the mouse granulosa cells were incubated with the following fluorescent probes: BODIPY TR-X Ryanodine (1 μM) to detect the ryanodine receptor [26,27], BODIPY-Red thapsigargin (1 μM) to localize the thapsigargin-sensitive Ca^2+^-ATPase (SERCA) [27], TO-PRO-1 iodide (50 nM) and DAPI (1 μg/ml) to stain nuclei [28], brefeldin A BODIPY 558/568 and BODIPY FL-conjugate isomer 1 (1 μM) to localize the ER and Golgi apparatus [29]. Fluorescent probes were incubated for 60–90 min at room temperature and washed 3 times with PBS for 5 min to minimize non-specific binding. To estimate non-specific binding of the fluorescent probes, cells were pre-incubated with 100 μM ryanodine, 100 μM thapsigargin, 100 μM brefeldin A according to [21]. Treated cells were visualized by confocal microscopy (see Immunochemistry Section).

### Ca^2+ ^dynamics by confocal microscopy

Ca^2+ ^mobilization was determined according to [21]. Briefly, mouse granulosa cells were loaded for 20–30 min at room temperature with 5 μM Fluo-4 AM in Krebs solution (KS; containing in mM): 150 NaCl, 1 KCl, 1 MgCl_2_, 1.8 CaCl_2_, 4 Glucose, 10 HEPES, pH adjusted to 7.4, with the addition of 5 mg/ml of BSA and 0.1 mg/ml of pluronic acid. The solution was filtered to eliminate particles. Cells were washed 3 times with KS to remove extracellular Fluo-4 AM and incubated for 10 min to complete de-esterification of the dye. The coverslips were mounted in a recording chamber and placed in a Nikon Eclipse E600 microscope. To apply the drugs, a home-made multichannel perfusion system was used. Perfusion of the solutions was continuous at 1 ml/min. Confocal images were obtained using a Nikon Plan-Flour × 20 multi-immersion objective (numerical aperture = 0.75) and captured at a resolution of 640 × 640 pixels in the scan mode (1 image/min). Photo-bleaching and photo-damage were minimized by reducing the laser power (95% attenuation). Fluorescent measurements were performed with an Argon laser (Fluo-4 AM was excited at 506 nm, and emission was collected at 526 nm).

### Fluorescent images analyzes and data fitting

Multiphotonic and confocal images (for control and experimental conditions) were obtained and analyzed in identical situation by LSM image and SIMPLE PCI software respectively. Immunochemical analyzes involved ≈ 20 cells, in which each cell was divided in 4–5 quadrant of similar dimensions (10 μm^2^) in both cytoplasmic and nuclear areas (n = 5). For analyses of Ca^2+ ^dynamics experiments, the complete nuclear and cytoplasmic areas of ≈ 200 cells were considered (n = 4).

Values are expressed as means ± S.E. Significance was tested by the Student's *t*-test. *P *< 0.05 was considered significant. Graphics were made with the Origin program (Version 5.0), and the images were processed with Photoshop program.

## Results

### Nuclear localization of the IP_3_R types 1, 2, and 3 in granulosa cells

**Figure 1:**
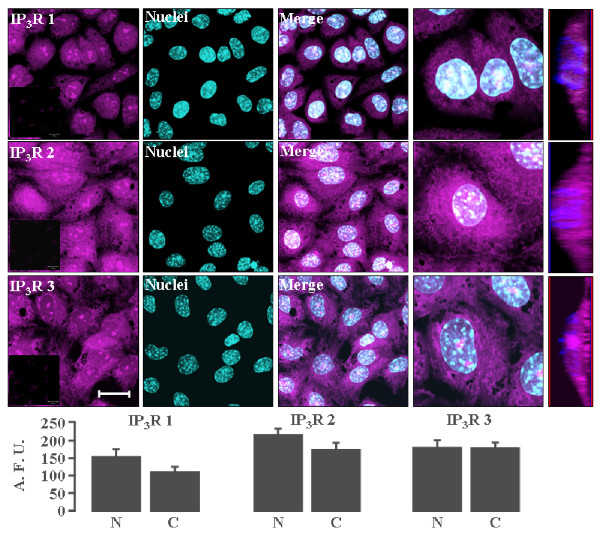
**Nuclear and cytoplasmic location of IP_3_R isoforms in mouse granulosa cells**. Confocal microscope images depict the subcellular location of IP_3_R isoforms detected by specific antibodies whereas nuclei were stained with DAPI. The first column shows the fluorescent signal elicited by the primary antibodies against IP_3_R types 1, 2, and 3, followed by the TX-Red-conjugated secondary antibody (Magenta color). No signal was detected when the primary antibodies were omitted (insets in first column). The second column depicts the fluorescent signal associated with the nuclear marker DAPI (Cyan color). The third column is the combination of the images in the first and second columns (merge), and the fourth column is a magnification of a selected sector of the merged image that strongly suggests the intranuclear presence of the three IP_3_R isoforms. The fifth column is a z-stack reconstruction of the fourth column (13 optics slices corresponding to 4 μm section). Histograms at the bottom show the quantification of the cytoplasmic (C) and nuclear (N) signals associated with each IP_3_R isoform. The measurements were obtained from the fluorescent signals within nuclei and cytoplasm using the LSM510 laser scanning microscope program. Scale bar corresponds to 20 μm. Results are expressed in arbitrary fluorescent units (AFU) and are the mean ± SEM of 5 independent experimental observations.

Commercial specific antibodies, tested in numerous previous reports [23,30], coupled to fluorescent moieties were used to determine the presence and intracellular distribution of IP_3_Rs in primary cultures of mouse granulosa cells by confocal microscopy (Figure 1) (analyzed cells ≈ 20; n = 5). All three types of IP_3_Rs were expressed at discernible levels, but in different patterns. Apparently, IP_3_R types 2 and 3 were the most abundant, whereas IP_3_R type 1 was detected in lesser amount, especially in the cytoplasm (Figure 1, images in the first column). Remarkably, the nuclear signal of all three IP_3_R isoforms was intense and well defined. Confirmation of the nuclear localization of the three IP_3_Rs isoforms was obtained by co-localization with DAPI, which is a specific nuclear marker (Nuclei and Merge columns in Figure 1). Images seen at higher magnification show an intricate cytoplasmic pattern for the three types of IP_3_Rs, with the signals for type 2 and 3 extended more towards the periphery of the cell (fourth column of Figure 1). In addition, magnified images clearly display nuclear signals for the three isoforms in both the nuclear envelope and the interior of the organelle. To further confirm the nuclear localization of the IP_3_R isoforms, the fifth column depicts a z-stack reconstruction (≈13 optical slices corresponding to 4 μm section) of the granulosa cells nuclei. It can be seen that the 3 types of IP_3_R show intranuclear localization, being more abundant the isoform 2 and 3. Therefore, primary cultures of mouse granulosa cells co-express all three IP_3_R isoforms, and unexpectedly, all of them are present within the nuclei. Low signal, barely detectable, was observed when the primary antibodies were omitted (Figure 1, insets in first column). To estimate the difference between the cytoplasmic and the nuclear IP_3_R distribution, a semi-quantitative analyzes was done (histograms at the bottom of Figure 1; cells analyzed ≈ 20; n = 5). IP_3_R type 1 and 2 showed an increased nuclear presence, whereas IP_3_R type 3 displayed similar signals in both compartments.

**Figure 2:**
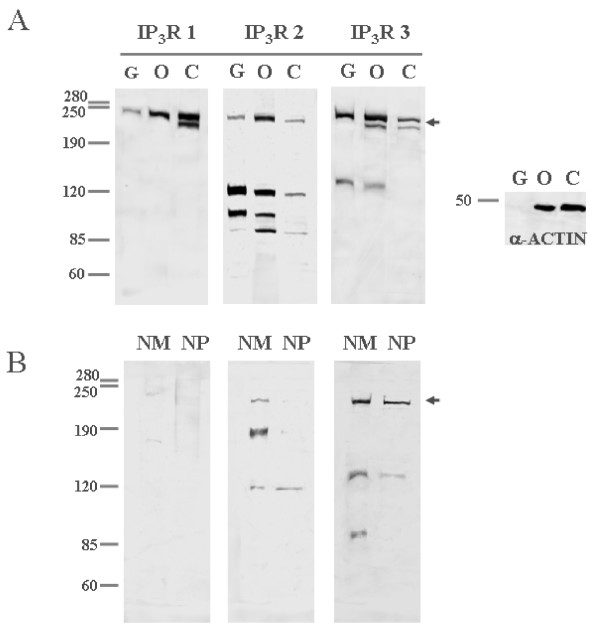
**Biochemical detection of IP_3_R isoforms by Western blot assays**. Arrows indicate the bands corresponding to the high molecular weight forms of the non-processed IP_3_Rs (≈230–250 kDa). Panel A. The presence of IP_3_R isoforms and low molecular weight variants in microsomal fractions from granulosa cells (**G**); tissues such as ovary (**O**), cerebellum (**C**), were obtained and analyzed by electrophoretic mobility. At the right side of Panel A the signal corresponds to α-actin (45 kDa). Panel B. The nuclear fractions (nuclear membranes (**NM**) and nucleoplasm (**NP**) from granulosa cells) were obtained and analyzed by electrophoretic mobility to detect the isoforms and low molecular weight variants of IP_3_Rs. Representative experiments of 3 independent observations.

Figure 2 panel A shows biochemical detection of IP_3_Rs isoforms by Western blot in reductive conditions, in granulosa cells (10^8 ^cells; n = 3), ovary and cerebellum (8 mice; n = 3). IP_3_R type 1 was detected in granulosa cells, ovary and cerebellum with a expected high molecular weight band (≈230–250 kDa [22,31,32]. In cerebellum, a second band (≈200 kDa) was also observed. IP_3_R type 2 was also seen in granulosa cells in a similar high molecular weight band (≈230–250 kDa), but in addition, three smaller bands were also detected (≈120, 100 and 90 kDa). The high molecular weight and smaller bands were also observed in ovary and cerebellum. The low molecular weight variants of IP_3_R type 2 might correspond to regulated proteolytic activity or to alternative mRNA processing [31-36]. IP_3_R type 3 in granulosa cells was detected as a high molecular weight band (≈230–250 kDa), but also in a band with lower molecular weight (≈130 kDa). IP_3_R type 3 was also present in ovary and cerebellum. At the right side of Figure 2 panel A, note that the signal corresponding to α-actin was detected in ovary tissue and not in granulosa cells treated with FSH *in vitro*, since the cytoskeleton of these cells does not contain this protein [37]. Taken together these results, it is shown that granulosa cells express the 3 IP_3_R isoforms, and in our experimental conditions granulosa cells are luteinized, and then they do not express α-actin.

Figure 2 panel B shows the presence of the three IP_3_R isoforms in isolated nuclei of granulosa cells (10^8 ^cells; n = 3), in two fractions: nuclear membranes (NM) and nucleoplasm (NP). For all three isoforms, a high molecular weight band (≈230–250 kDa) corresponding to the complete form of IP_3_R was observed in the NM fraction. However the band of IP_3_R type 1 was clearly fainter than the other two types. Two smaller variants of IP_3_R types 2 and 3 were also detected in this fraction. The NP fraction showed no signal for the complete IP_3_R type 1; in contrast a weak band corresponding to IP_3_R type 2 whereas the band for type 3 was stronger. Low molecular weight variant was clearly detected for types 2 and 3.

### Cytoplasmic and nuclear Ca^2+ ^mobilization in granulosa cells

**Figure 3:**
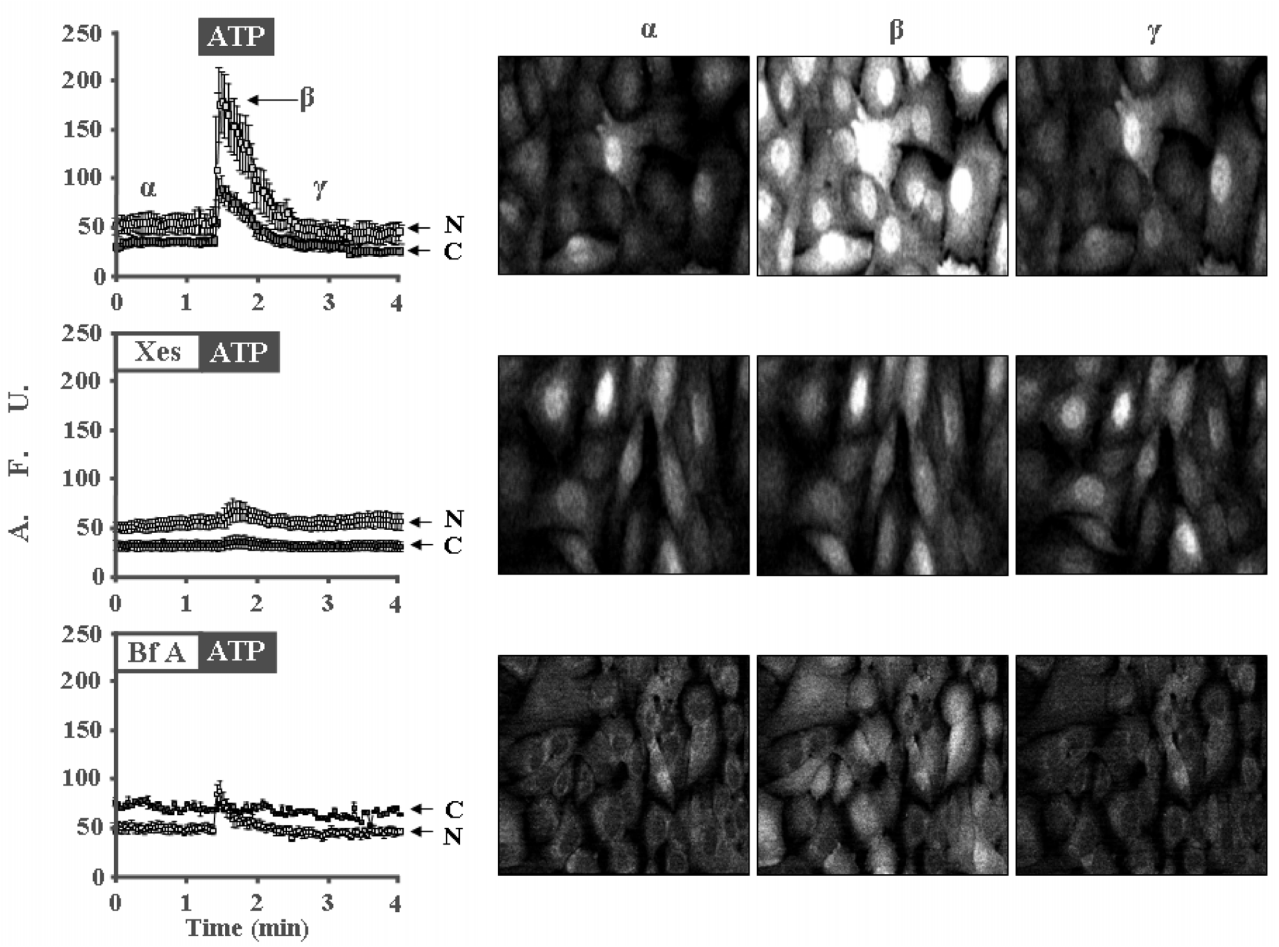
**ATP-induced Ca^2+ ^mobilization in the nucleus and cytoplasm of mouse granulosa cells**. Cells were incubated with Fluo-4 AM (5 μM) and analyzed by confocal microscopy. Each image represents the temporal changes in [Ca^2+^]i of a set of nearly 200 granulosa cells. Plots in the left column show the temporal pattern of fluorescent fluctuations of each experiment. Images were acquired every second, and the signals are expressed in arbitrary fluorescent units (AFU). N = nucleus and C = cytoplasm. The period of ATP (50 μM) delivery is indicated in the black box, whereas application of xestospongin C (Xes) (5 μM for 15 min) and brefeldin A (Bf A) (5 μg/ml for 2 h) is depicted in white boxes. Greek letters represent Ca^2+ ^mobilization before (α), during (β), and after (γ) ATP application. In the plot, light symbols indicate the temporal pattern of Ca^2+ ^mobilization within the nucleus, whereas dark symbols correspond to the variation of cytoplasmic Ca^2+^. First block: Representative experiment of Ca^2+ ^mobilization by ATP. Second and third blocks: Inhibitory action of pre-treatment with xestospongin C and brefeldin A on the nuclear and cytoplasmic ATP-induced Ca^2+ ^mobilization. Representative of 4 independent experiments.

To examine the functionality of the nuclear Ca^2+^-handling proteins detected in the mouse granulosa cells, ATP-induced Ca^2+ ^mobilization was assessed by confocal microscopy (analyzed cells = 800; n = 4). Application of ATP (50 μM) to the assay promoted Ca^2+ ^mobilization by activation of purinergic G-protein-coupled receptor P2Y2 in mouse granulosa cells [3,21]. The Fluo-4 associated signal was higher in the nuclei than in the cytoplasm, even in basal, non-stimulated conditions (Figure 3, first block, and column α). However, this fact could be due to nuclear milieu influence over the fluorescent signal, and not necessarily represent an elevated Ca^2+ ^level within the nucleus. Upon ATP stimulation, the Ca^2+ ^transient elicited in the nucleus was higher than the one recorded in the cytoplasm (Figure 3, first block, and column β). After the transient, intracellular Ca^2+ ^returned to basal levels, but the nuclear fluorescent signal remained higher than in the cytosol (Figure 3, first block, and column γ). Treatment with the IP_3_R non-competitive inhibitor xestospongin C (5 μM for 15 min) prevented the ATP-induced Ca^2+ ^mobilization in both compartments, without changes in the basal Ca^2+ ^(Figure 3, second block, columns α, β, and γ). Interestingly, the ATP-induced Ca^2+ ^transient in the presence of the antibiotic brefeldin A (5 μg/ml for 2 h) showed a differential response comparing the cytoplasmic and nuclear compartments. In this case, Ca^2+ ^mobilization was completely suppressed in the cytoplasm, but in the nucleus the brefeldin A effect was only partial, and a net Ca^2+ ^mobilization could be observed (Figure 3, third block, and column β). In addition, brefeldin A treatment promoted a decrease in the basal Fluo-4 associated-signal within the nucleus (Figure 3, third block, columns α, β and γ). These data support the notion that the nuclei in the granulosa cells, as the nuclei of others cellular systems, also have the capacity to mobilize Ca^2+ ^[14].

### Ryanodine receptor, thapsigargin-sensitive Ca^2+^-ATPase, and endomembranes are also present in the nuclei of granulosa cells

**Figure 4:**
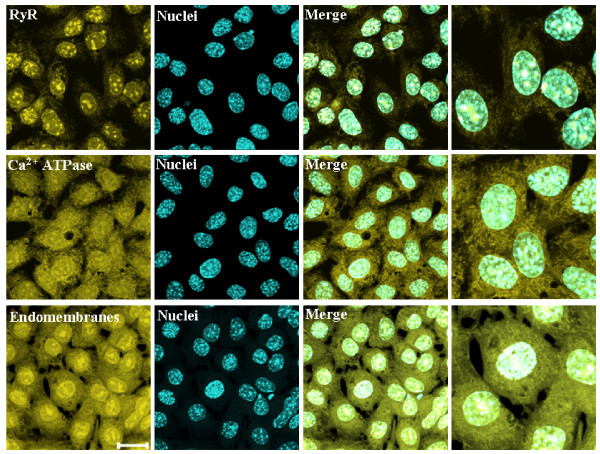
**Subcellular location of ryanodine receptor, thapsigargin-sensitive Ca^2+^-ATPase, and endomembranes in mouse granulosa cells**. The first block shows the intracellular location of the ryanodine receptor visualized by BODIPY FL-ryanodine. The second block depicts the intracellular location of thapsigargin-sensitive Ca^2+^-ATPase using BODIPY-FL thapsigargin. The third block shows the distribution of endomembranes by means of the BODIPY-FL conjugate of brefeldin A. All fluorescent probes were assayed at 1 μM (Yellow color). The second column shows the signal of the nuclear marker DAPI (Cyan color), whereas the third column depicts the merging the first and second columns. The fourth column is a magnification of a selected sector of the merge image. The results strongly suggest the intranuclear presence of all the Ca^2+^-handling proteins and the endomembranes in the mouse granulosa cells. Scale bar corresponds to 20 μm. Representative of 5 independent experiments.

By means of specific fluorescent probes, the subcellular localization of RyR, SERCA, and the network of endomembranes in granulosa cells were investigated (n = 5). The results are shown in Figure 4. RyR formed clusters throughout the cytosol, and its expression within the nucleus was demonstrated by the coincidence of the fluorescent ryanodine derivative with the nuclear marker DAPI. The magnified image depicts more clearly the nuclear and perinuclear localization of the RyR. The fluorescent signal associated with thapsigargin showed the presence of SERCA in the cytoplasm as thread-like structures, and as with the RyR, the nuclear localization of SERCA was also evident from the coincidence with the DAPI signal. With higher magnification, the widespread distribution of SERCA throughout the entire cytoplasm as well as its intranuclear localization can be appreciated. Additionally, mouse granulosa cells were incubated with a fluorescent derivative of brefeldin A, a fungal metabolite that binds selectively to endomembranes, namely endoplasmic reticulum and the Golgi complex [29]. As with RyR and SERCA, the endomembrane marker was present in the cytoplasm, where it formed numerous clusters, and also within the nucleus. Co-localization with DAPI further supported the intranuclear localization of fluorescent brefeldin A, suggesting the presence of a membranous system within the nucleus (possibly the nucleoplasmic reticulum) in mouse granulosa cells. These characteristics are apparent in the magnified image. Taken together, these results indicate that the nuclei of granulosa cells in culture conditions express the principal Ca^2+^-handling proteins, as well as membranal structures in the nucleoplasmic compartment. These findings are similar to results reported in other cellular systems [10-14].

### Isoforms of IP_3_Rs in mouse ovary

**Figure 5:**
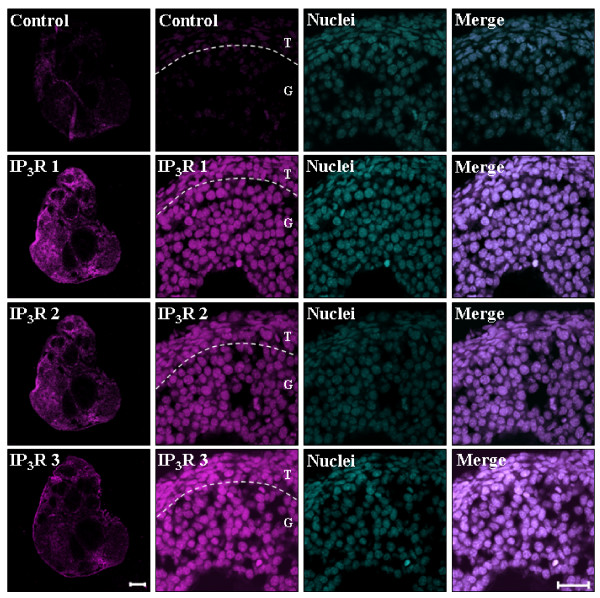
**Detection of IP_3_R isoforms in mouse ovarian tisuue**. Ovarian tissue was incubated with specific antibodies against IP_3_R types 1, 2, and 3, as well as with TO-PRO-1 iodide (50 nM) to label nuclei. Control images done in absence of primary antibody are at the top of columns 1 and 2. The first column shows entire ovarian structures, whereas the second column depicts an enhanced image delimiting granulosa (G) and theca (T) cells (Magenta color). The third column shows nuclear signal associated with TO-PRO-1 (Cyan color), and the fourth column represents the convergence of signals between IP_3_Rs and nuclear structures (merge). Scale bar for ovary slices corresponds to 100 μm, and for granulosa cells to 20 μm. These results provide evidence for the intranuclear presence of all the IP_3_R isoforms in the mouse granulosa cells *in situ*. Representative of 3 independent experiments.

To eliminate possibly that our results reflected an artifact related to the culture conditions, we tested if the three IP_3_R isoforms were also observed within the granulosa cells nuclei in histological slices of mouse ovarian tissue (n = 3). Figure 5 shows the signals associated with the same isoform-specific antibodies against the IP_3_R types 1, 2, and 3 that were used in the assays with granulosa cells *in vitro*. Consistent with the previous results, the three IP_3_R isoforms were also detected in the granulosa cell nuclei *in situ *when these cells are still part of the ovary, which are located between the oocyte and the theca cells. The specificity of the experiments was confirmed by the very low unspecific signal seen when only the secondary antibodies were used (Figure 5, Control). These results confirm previous reports in which the three IP_3_R isoforms were detected in ovary [19,20].

The distribution of the three types of IP_3_R was similar along the slices of ovarian tissue. The nuclear expression of the three IP_3_R isoforms was evident from their co-localization with the nuclear marker TO-PRO 1 iodide. Analogous results were also obtained with the theca cells. These data indicate that mouse granulosa cells express, both in culture and *in situ*, all three types of IP_3_Rs with a cytoplasmic and nuclear localization.

## Discussion

### Expression and subcellular localization of IP_3_R isoforms in granulosa cells

Granulosa cells, like many other cellular types, contain at least two forms of Ca^2+ ^release channels: RyR and IP_3_R [21]. Both channels are localized in the endoplasmic reticulum membranes, but also within the nuclear structure (Figures 1 and 4). Our data show that granulosa cells express the three IP_3_R isoforms (Figure 1). Detection of the IP_3_R isoforms was similar in both experimental conditions tested: in primary cultures (Figures 1 and 2), and in ovary slices (Figure 5). Certainly, we do not know if the immuno-detected IP_3_Rs are homo or heterotetramers. However, we have evidence that granulosa cells express low molecular weight variants of IP_3_R types 2 and 3 which could result from regulated proteolysis or mRNA splicing (Figure 2) [31-36]. Hence, granulosa cells have the potential to display a great variety of Ca^2+ ^signaling responses based in the molecular diversity of IP_3_Rs forms.

The occurrence of at least two different isoforms of IP_3_R has been reported in several cellular types, such as hepatocytes (types 1 and 2) [38], lung (types 2 and 3) [32], colonic epithelium (types 2 and 3) [39], and deep cerebellar nuclei (types 1 and 3) [40]. The congregation of the three isoforms of IP_3_R in a unique cellular population is less frequent. Besides the finding from this study in mouse granulosa cells, the three types of IP_3_R have been reported to coexist in rat bile duct epithelial cells or cholangiocytes [41] and in bovine adrenal chromaffin cells [42].

When two or three IP_3_R isoforms are expressed in the same cell, they are usually distributed in different subcellular regions: For example, IP_3_R type 3 is localized in the apical section of cholangiocytes and non-pigmented epithelium cells, whereas the other isoforms are present in the rest of the cell structure, especially in the basolateral region [38,43]. In the case of the mouse granulosa cells, we did not observe any preferential subcellular location for the IP_3_R isoforms: The signal from the three types of IP_3_Rs occurs throughout most of cytoplasm, presumably along the granulosa cells endomembranes, with a less intense signal for the type 1 (Figure 1). The identity of the organelles in which the IP_3_R isoforms were detected will be treated later.

In heterologous systems (Sf9 insect cells) the expression of each isoform of the IP_3_Rs presents the same Ca^2+ ^gating and similar ionic conductance [44]. However, they differ in their sensitivity to IP_3_, intracellular Ca^2+^, and ATP: type 1 shows medium IP_3_-affinity, high ATP-affinity and low Ca^2+ ^affinity. IP_3_R type 2 has a high IP_3_-affinity, a medium Ca^2+ ^affinity, and is ATP independent. IP_3_R type 3 shows a low IP_3_-affinity, a low ATP-affinity, and a high Ca^2+ ^affinity [44].

IP_3_R type 3 is related to the triggering of Ca^2+ ^waves in diverse tissues, such as polarized epithelia [38], as well as to act as an apoptotic mediator in different cellular systems [45]. As to granulosa cells, it is not clear if they can be considered polarized, but some authors postulate a directionality in their function when, as a cellular population, the granulosa cells are surrounding the oocyte. For example, the handling of intracellular Ca^2+ ^in the granulosa cells close to the theca is different from the granulosa cells close to the oocyte [46]. However, at least in the ovarian slice, there was no indication of the existence of a cellular sub-population with regard to the three IP_3_R isoforms. As to the role of Ca^2+ ^release channels in promoting Ca^2+ ^signaling, it was reported that the activity of RyR was necessary for ATP-induced Ca^2+ ^mobilization in mouse granulosa cells [21]. More systematic studies are needed to define the precise role of the three IP_3_R isoforms during spontaneous and ligand-induced Ca^2+ ^transients in granulosa cells. Although it is well documented that granulosa cells are prone to apoptosis according to hormonal and nutritional factors [47], the exact role of IP_3_R type 3 or the other two isoforms has not be substantiated during this process when this cell population is luteinized.

### Nuclear location of IP_3_R isoforms in granulosa cells

IP_3_Rs have long been known to be present within the sarco-endoplasmic reticulum membranes of many cellular types and tissues (for review see [48]). However, growing evidence indicates the existence of the IP_3_Rs in other intracellular organelles such as the Golgi apparatus [49], plasma membrane [50,51], and nucleus [8-18,52]. Interestingly, in several cell types the IP_3_R has been detected in the nuclei, specifically in the nuclear envelope membranes, but also within membranous reticular structures known as the nucleoplasmic reticulum [17]. This intranuclear system is able to store and release Ca^2+ ^in the same way as the endoplasmic reticulum does in the cytoplasm [17,53]. To our knowledge, the results reported here are the first to show that the nuclei of granulosa cells contain all three isoforms of the IP_3_R. The intranuclear distribution of each isoform of the IP_3_Rs is variable; for example, types 1 and 3 were preferentially localized in the inner nuclear membrane of skeletal muscle myocytes [54]. In contrast, isoform 1 in the nuclei of bovine aortic endothelial cells, bovine adrenal glomerulosa cells, COS-7 cells [55], and ventricular myocytes [56] was not confined to the nuclear envelope, but distributed uniformly within the nucleus. Type 2 was detected forming part of the nucleoplasmic reticulum in rat hepatocytes [17]. The exact role of each IP_3_R isoform in the generation and kinetics of Ca^2+ ^transients in the nucleus and cytoplasm of all these cells remains to be explored.

Nuclei in the granulosa cells contain the most important elements to accomplish Ca^2+ ^release and uptake: besides IP_3_Rs, we detected positive and specific signals for RyR, thapsigargin-sensitive SERCA, and endomembranes (Figures 1 and 4). It has been postulated that the cellular pattern of Ca^2+ ^dynamics depends on the isoforms of the IP_3_Rs, RyRs, and SERCAs present in the different organelles [11]. Indeed, the results in Figure 3 indicate that cytoplasm and nuclei differ in their capacity for ATP-induced Ca^2+ ^mobilization due to differences in the extent to which brefeldin A alters Ca^2+ ^dynamics in the two compartments. Nuclear Ca^2+ ^has been shown to be specifically and autonomously mobilized in a great variety of other cellular types [53]. Questions arise regarding the potential functions of Ca^2+ ^fluctuations within the nucleus. Several reports have shown that nuclear Ca^2+ ^can control the transcriptional activity of certain genes [57], protein transport across the nuclear envelope [58], and translocation of protein kinases [17,59]. More experiments are needed to determine how these ion channels and metabolic pumps are coordinated to enable nuclear Ca^2+ ^transients in synchronization with cytosolic Ca^2+ ^dynamics in granulosa cells.

### IP_3_R isoforms in granulosa cells: In situ and in culture conditions

Granulosa cells are part of an ovarian complex where different follicular cell types are congregated to promote the development and maturation of the oocyte. Within this complex, granulosa cells are found in a precise location between the oocyte and the theca cells. It is a controversial issue if granulosa cells change their physiological characteristics when they are dissected and placed in culture conditions [60]. However, there are numerous reports that consider granulosa cells *in vitro *to be a suitable experimental system. Hence, studies of granulosa cells properties in culture conditions regarding hormonal action, signal transduction, and cell differentiation are common [61].

The findings of this study indicate that the presence of the three IP_3_R isoforms in the cytoplasmic and nuclear membranes is comparable in granulosa cells maintained in *in vitro *conditions with granulosa cells as part of the *in vivo *histological architecture; however, this conclusion must be confirmed. There are three potential interpretations of these observations: First, the expression of the three IP_3_R isoforms is not an artifact associated with the manipulation of granulosa cells when they are put in culture. Second, it is highly probable that granulosa cells express the three IP_3_R types *in vivo*, which would indicate a potential enriched repertoire in the intracellular Ca^2+ ^dynamics of these ovarian endocrine cells. Third, granulosa cells are among the cellular types that express consistently the three isoforms of the IP_3_R, and the only type known so far that expresses all of them within the nucleus.

## Conclusion

1) We are reporting for the first time that one type of murine granulosa cell expresses in cytoplasmic endomembranes and nucleus all three types of IP_3_Rs. 2) Both compartments mobilized Ca^2+ ^in response to ATP, but the nucleus was less sensitive to the inhibitory action of brefeldin A. 3) Studies on intracellular Ca^2+ ^mobilization in mouse granulosa cells should provide further information regarding the molecular and cellular events that are relevant for the participation of somatic cells in regulating the ovarian follicular development.

## Competing interests

The authors declare that they have no competing interests.

## Authors' contributions

MDM participated in designing the study, in the analysis and discussion of the results and drafted critical revision of the manuscript. PdlRS participated in the initial immunofluorescent experiments. ABJE and ROA in the experimental design of the ovary histology. VMT designed the study, performed most of the experiments, and participated in the analysis and discussion of the results and drafted the manuscript. All authors read and approved the final manuscript.
